# Allopregnanolone Decreases Evoked Dopamine Release Differently in Rats by Sex and Estrous Stage

**DOI:** 10.3389/fphar.2020.608887

**Published:** 2021-01-14

**Authors:** Ana Paula S. Dornellas, Giovana C. Macedo, Minna H. McFarland, Alexander Gómez-A, Todd K. O’Buckley, Claudio Da Cunha, A. Leslie Morrow, Donita L. Robinson

**Affiliations:** ^1^ Bowles Center for Alcohol Studies, University of North Carolina, Chapel Hill, NC, United States; ^2^ Laboratório de Fisiologia e Farmacologia do Paraná, Departments of Pharmacology and Biochemistry, Universidade Federal do Paraná, Curitiba, Brazil; ^3^ Department of Psychobiology, Universidade Federal de São Paulo, UNIFESP, São Paulo, Brazil; ^4^ Department of Psychiatry, University of North Carolina, Chapel Hill, NC, United States; ^5^ Department of Pharmacology, University of North Carolina, Chapel Hill, NC, United States

**Keywords:** neurosteroid, allopregnanolone, progesterone, voltammetry, dopamine, nucleus accumbens

## Abstract

Mesolimbic dopamine transmission is dysregulated in multiple psychiatric disorders, including addiction. Previous studies found that the endogenous GABAergic steroid (3α,5α)-3-hydroxy-5-pregnan-20-one (allopregnanolone) modulates dopamine levels in the nucleus accumbens and prefrontal cortex. As allopregnanolone is a potent positive allosteric modulator of GABA_A_ receptors, and GABA_A_ receptors can regulate dopamine release, we hypothesized that allopregnanolone would reduce phasic fluctuations in mesolimbic dopamine release that are important in learning and reward processing. We used fast-scan cyclic voltammetry in anesthetized female and male rats to measure dopamine release in the nucleus accumbens evoked by electrical stimulation of the ventral tegmental area, before and after administration of allopregnanolone. Allopregnanolone (7.5–25 mg/kg, IP) reduced evoked dopamine release in both male and female rats, compared to β-cyclodextrin vehicle. In males, all doses of allopregnanolone decreased dopamine transmission, with stronger effects at 15 and 25 mg/kg allopregnanolone. In females, 15 and 25 mg/kg allopregnanolone reduced dopamine release, while 7.5 mg/kg allopregnanolone was no different from vehicle. Since allopregnanolone is derived from progesterone, we hypothesized that high endogenous progesterone levels would result in lower sensitivity to allopregnanolone. Consistent with this, females in proestrus (high progesterone levels) were less responsive to allopregnanolone than females in other estrous cycle stages. Furthermore, 30 mg/kg progesterone reduced evoked dopamine release in males, similar to allopregnanolone. Our findings confirm that allopregnanolone reduces evoked dopamine release in both male and female rats. Moreover, sex and the estrous cycle modulated this effect of allopregnanolone. These results extend our knowledge about the pharmacological effects of neurosteroids on dopamine transmission, which may contribute to their therapeutic effects.

## Introduction

Neuroactive steroids (neurosteroids) are compounds synthesized *de novo* in neurons that modulate both gene expression and neuronal excitability, the latter through interactions with neurotransmitter receptors (for review, see Porcu et al., 2016; Paul et al., 2020). Neurosteroid levels in serum and brain are dysregulated in multiple psychiatric disorders (e.g., Rapkin et al., 1997; Brambilla et al., 2003; Hellgren et al., 2014; Frau et al., 2020; Hantsoo and Epperson, 2020). Moreover, allopregnanolone, a metabolite of progesterone and a GABAergic neurosteroid, has emerged as a clinically beneficial therapeutic (Milivojevic et al., 2016; Bixo et al., 2017; Meltzer-Brody et al., 2018). For example, progesterone reduced cocaine craving in individuals with cocaine-use disorder (Fox et al., 2013) and this effect was highly correlated with circulating allopregnanolone (Milivojevic et al., 2016).

The mesolimbic dopamine pathway extends from the ventral tegmental area (VTA) to limbic and cortical areas and is involved in complex processes, such as decision making and motivated behavior, and its dysregulation contributes to several psychiatric disorders, including addiction (Volkow et al., 2017). Some strategies of psychoactive treatment aim to directly modify dysregulated dopamine transmission (e.g., German et al., 2015); however, dopamine itself is a difficult therapeutic target due to potential side effects, particularly those affecting movement and motivation (e.g., Kaar et al., 2019). Instead, indirect modulation of dopamine through γ-aminobutyric acid type A (GABA_A_) receptors (Nikolaus et al., 2018; Lopes et al., 2019; Kramer et al., 2020) may prove to be a useful strategy to regulate dopamine. Benzodiazepines can modulate dopamine (Takada et al., 1993; Finlay et al., 1995; Gómez-A et al., 2017; Brodnik et al., 2019), but these medications can also have both acute and chronic side effects, as well as high dependence and abuse potential (Licata and Rowlett, 2008; Votaw et al., 2019). Previous studies found that neurosteroids can modulate dopamine concentrations. For example, five days of progesterone administration enhanced the alcohol to dose-dependently modulate extracellular dopamine concentrations in the prefrontal cortex (Dazzi et al., 2002). Moreover, the endogenous neurosteroid derived from the hormone deoxycorticosterone, 3α,21-dihydroxy-5α-pregnane-20-one (THDOC) blunted stress-induced increases in dopamine tissue content in the prefrontal cortex (Grobin et al., 1992). Furthermore, intracerebroventricular allopregnanolone also increases tonic dopamine transmission in the nucleus accumbens (NAc) (Rouge-Pont et al., 2002) and modulates tissue content of dopamine (Laconi et al., 2007). However, no studies to date have assessed the regulation of phasic dopamine release–brief dopamine fluctuations resulting from burst-firing–by neurosteroids. This aspect of dopamine release is of interest due to its role in reward-associated learning and addiction (Wightman and Robinson, 2002; Schultz, 2007; Schultz, 2016; Volkow et al., 2017).

As allopregnanolone is inhibitory due to its allosteric actions at GABA_A_ receptors, and based on the above evidence that drugs that enhance GABA_A_ receptor activity reduce dopamine release, we hypothesized that it would inhibit phasic dopamine release. To test this, we used fast-scan cyclic voltammetry, a technique that offers the temporal, spatial, and chemical resolution required to assess fast dopamine release events (Robinson et al., 2003; Robinson et al., 2008). We measured phasic fluctuations in dopamine release evoked by electrical stimulation of the VTA in anesthetized male and female rats before and after intraperitoneal injections of allopregnanolone or β-cyclodextrin vehicle. Progesterone, the precursor to allopregnanolone, has great translational relevance due to its clinical availability when compared to allopregnanolone, and so we also examined its effects on evoked mesolimbic dopamine release. We predicted that both allopregnanolone and progesterone would decrease VTA-evoked dopamine release in the NAc.

## Materials and Methods

### Animals

Adult male (N = 30, 297 ± 4 g at experiment) and female (N = 27, 216 ± 2 g at experiment) Sprague–Dawley rats (Envigo; Frederick, MD) were used in the present study. Animals were housed in a temperature-controlled environment (21 ± 1°C) with 12-h light/dark cycles and *ad libitum* access to food and water. Males were housed in groups of 2–3 animals per cage and females in groups of 2–4 per cage. A subset of female rats (n = 19) were assessed for estrous cycle stage by cell morphology after vaginal lavage with 20 µl of saline, immediately after the experiment. Data collection occurred between 1200 and 1700 h each experimental day, for all the male and female animals. Vaginal lavage occurred at the end of the experiment – typically between 1400–1500 h, but occasionally as late as 1700 h. All procedures were approved by the Institutional Animal Care and Use Committee of University of North Carolina at Chapel Hill.

### Drugs

Allopregnanolone, purchased from the late Dr. R. H. Purdy (formerly of the Veterans Medical Research Foundation, San Diego, CA, United States), was added to 45% hydroxypropyl-β-cyclodextrin (Acros Organics, ThermoFisher Scientific, Waltham, MA, #297565000) in water, vortexed, mixed in an ultrasonic water bath for 15–20 min, then stored while continuously stirring at 4°C, and kept for a maximum of two days after preparation. Progesterone (Steraloids, Newport, RI, #Q2600) was added to the 45% hydroxypropyl-β-cyclodextrin solution, vortexed, then stored while continuously stirring at 4°C. The doses of allopregnanolone and progesterone used in the study were selected as doses with neuroactive (anti-seizure) effects but minimal sedative effects in rats (Lonsdale et al., 2006; Wu and Burnham, 2018).

### Surgery

Rats were anesthetized with urethane (50% w/w in saline; 1.2–1.5 g/kg) and secured in a stereotaxic frame on a heated pad. Urethane was chosen due to modest effects on multiple neurotransmitter-gated ion channels (Hara and Harris, 2002 Maggi and Meli, 1986) and the lack of effect on dopamine clearance *in vivo* (Garris et al., 1997; Sabeti et al., 2003). Anterior–posterior (AP), medial–lateral (ML), and dorsal–ventral (DV) positions refer to bregma, and coordinates were obtained from a rat brain atlas (Paxinos and Watson, 1998). A bipolar stimulating electrode (Plastics One, Roanoke, VA, United States, polished tips, 1 mm apart) was placed above the VTA (AP: −5.3, ML: −0.9, DV: −8.1), a recording electrode was placed in the NAc (AP: +1.6, ML: −2.0, DV: −6.2) and an Ag/AgCl reference electrode was implanted in the contralateral cortex. The recording electrode consisted of a single carbon fiber (T650, Thornel/Cytec Industries Inc., Woodland Park, NJ, United States; 6-μm diameter) sealed in a glass capillary (600 µm O.D.). The carbon fiber extended 80–120 µm from the glass seal and was soaked for at least 10 min in isopropyl alcohol to clean the carbon fiber (Bath et al., 2000).

### Fast Scan Cyclic Voltammetry

Voltammetric parameters, electrical stimulation parameters, and data acquisition were controlled by a computer using LabVIEW instrumentation software (National Instruments, Austin, TX) as previously described (Shnitko et al., 2016). In brief, voltammetric recordings were made at the carbon-fiber microelectrodes by applying a triangle waveform potential, ramping from −0.4 V to +1.3 V and back to −0.4 V, at a scan rate of 400 V/s. The triangle-waveform was applied at 60 Hz for the first 20 min to condition the electrode, after which the application was reduced to 10 Hz. Dopamine release was evoked by electrical stimulation of the VTA consisting of 24 biphasic, square-wave pulses (60 Hz, 125 μA, 2 ms/phase).

After electrodes were initially placed, dopamine neurons were activated every 2–5 min via electrical stimulation to the VTA and dopamine release was detected at the carbon-fiber electrode. Current was confirmed to be due to oxidation of dopamine and reduction of the ortho-quinone via the background-subtracted cyclic voltammogram. Evoked dopamine release was optimized by moving the stimulating and/or carbon-fiber electrodes ventrally at 100-µm increments. The average final DV placement of the stimulating electrode in the VTA was 8.3 (range: 8.1–8.9 mm) in females and 8.5 (range: 8.1–8.9 mm) in males. The average final DV placement of the carbon-fiber electrode in the NAc was 6.5 mm (range: 6.2–7.1 mm) in females and 6.8 (range: 6.2–7.6 mm) in males. The experimental recording began when evoked dopamine release reached a minimum of 2.5 nA and when the signal-to-noize ratio was higher than 25. Thereafter, dopamine release was evoked at 5-min intervals, and an average of nine electrically-evoked signals were collected before starting the experiment in order to confirm stability of the signal. Next, to ensure that injections did not alter the dopamine signal and that the signal was stable, animals received two saline injections (IP), 15 min apart. Finally, animals received allopregnanolone, progesterone, or vehicle (IP), followed by 60 min of electrochemical recording. The average calibration factor used to estimate dopamine concentration from current was 1 nA = 0.103 ± 0.017 µM, extracted from a library of 107 *in vivo* electrodes used in the lab that were calibrated in a flow-cell with 1 µM DA in TRIS buffer (Logman et al., 2000; Robinson et al., 2009).

### Statistical Analysis

Evoked dopamine was characterized by calculating [DA]_max_ (maximum dopamine concentration) and T½ (the time for dopamine to clear to half maximum concentration) as measures of dopamine release and clearance, respectively (Yorgason et al., 2011; Shnitko et al., 2016). As the effect of neurosteroids on dopamine release was expected to be inhibitory, it was critical that we used stringent inclusion criteria of electrode stability, so that reductions in signal could be attributed to neurosteroid action rather than to time. For inclusion of data from a rat, the nine evoked dopamine signals prior to the injection of neurosteroids or vehicle (45 min, including the two saline injections) were required to vary <15% and exhibit a signal-to-noize ratio >25.

To broadly compare evoked dopamine release and clearance at baseline by sex or by estrous cycle stage, we used Mann-Whitney U tests or Kruskal-Wallis one-way ANOVA on ranks due to the non-normal distribution of the data. For these comparisons, we used raw (not normalized) [DA]_max_ and T½ values. Neurosteroid effects on evoked dopamine over time were assessed using repeated-measures (RM) ANOVA, followed by Holm-Sidak post-hoc comparisons, as appropriate. For these analyses, we used raw T½ values and normalized [DA]_max_ values. Specifically, the raw [DA]_max_ data did not pass the Shapiro-Wilk normality test, and to account for individual variability due to electrode placement or length, [DA]_max_ data were normalized within each rat to the evoked dopamine levels during the saline injections (six evoked signals immediately preceding the neurosteroid or vehicle injections); the transformed data passed the normality test.

For statistical analysis, data were pooled into 15-min bins (three evoked signals/bin), resulting in seven time points (three pre-injection, four post-injection). For the animals that received allopregnanolone (Allo) or vehicle, the pre-injection bins were indicated as BL (basal), Sal (saline) 1, and Sal 2, and the post-injection as Allo 1, Allo 2, Allo 3, and Allo 4. For those that received progesterone (Prog), the bins were BL, Sal 1 and Sal 2, followed by Prog 1 to Prog 4. Effect sizes were considered small, medium, or large if they corresponded to partial *η* ^2^ of at least 0.0099, 0.0588, and 0.1379, respectively, based on values of ƒ as described by Cohen (Cohen, 1988). Analyses were calculated using SigmaPlot for Windows v. 11 (Systat Software, Inc. San Jose, CA), graphs were made using GraphPad Prism 8.0.0 (GraphPad Software, San Diego, CA), and results are shown as mean ± SEM.

## Results

### Allopregnanolone Reduced Evoked Dopamine Release Into the NAc Differently in Male and Female Rats

**FIGURE 1:**
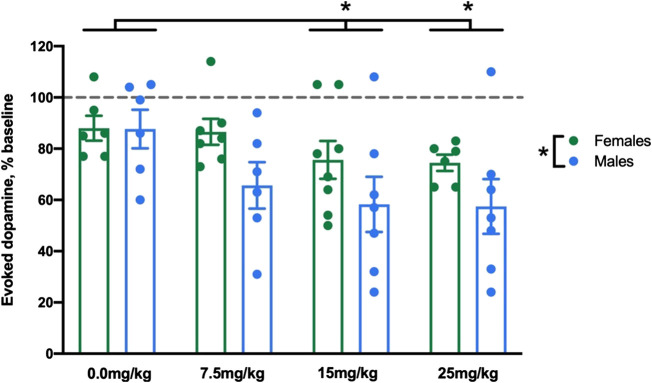
Allopregnanolone reduced evoked dopamine release at multiple doses in both males and females. We compared the effect of allopregnanolone and vehicle at 45–60 min post-injection in male and female rats. Evoked dopamine release is displayed as percent baseline (dotted line). We found a main effect of sex, in that males showed lower evoked dopamine than females when collapsed across dose. We also observed a main effect of dose, in that 15 and 25 mg/kg allopregnanolone (collapsed across sex) induced significantly lower evoked dopamine concentrations than vehicle (n = 6–8 per group).

Allopregnanolone (or vehicle) was administered at 7.5, 15, and 25 mg/kg to separate groups of male and female rats. We first compared baseline dopamine signals between males and females and found no difference in [DA]_max_ or T½; these data are reported in detail in the Supplementary Material. Next, as an initial analysis of the effects of dose by sex, we analyzed [DA]_max_ (normalized to saline injection, as described in *Statistical Analysis*) at the final time bin (45–60 min post-injection, bin Allo 4) representing the maximal effect of allopregnanolone. Allopregnanolone reduced evoked dopamine release at multiple doses in both males and females (Figure 1). The 2-way ANOVA revealed significant main effects of dose (F_3,45_ = 3.1, $\eta_{p}^{2}=0.17$, *p* < 0.05) and sex (F_1,45_ = 6.0, $\eta_{p}^{2}=0.12$, *p* < 0.05), with no significant interaction (F_3,45_ = 0.6, *p* = 0.61). Post-hoc comparisons following on the main effect of dose found that, collapsed across sex, both the 15 and 25 mg/kg doses were significantly different from vehicle (*p* < 0.05). The main effect of sex indicated that, collapsed across dose, males exhibited larger reductions in [DA]_max_ after injections than females. Due to this sex difference, we next analyzed the dose-dependent effects of allopregnanolone over time separately within males and females.

**FIGURE 2:**
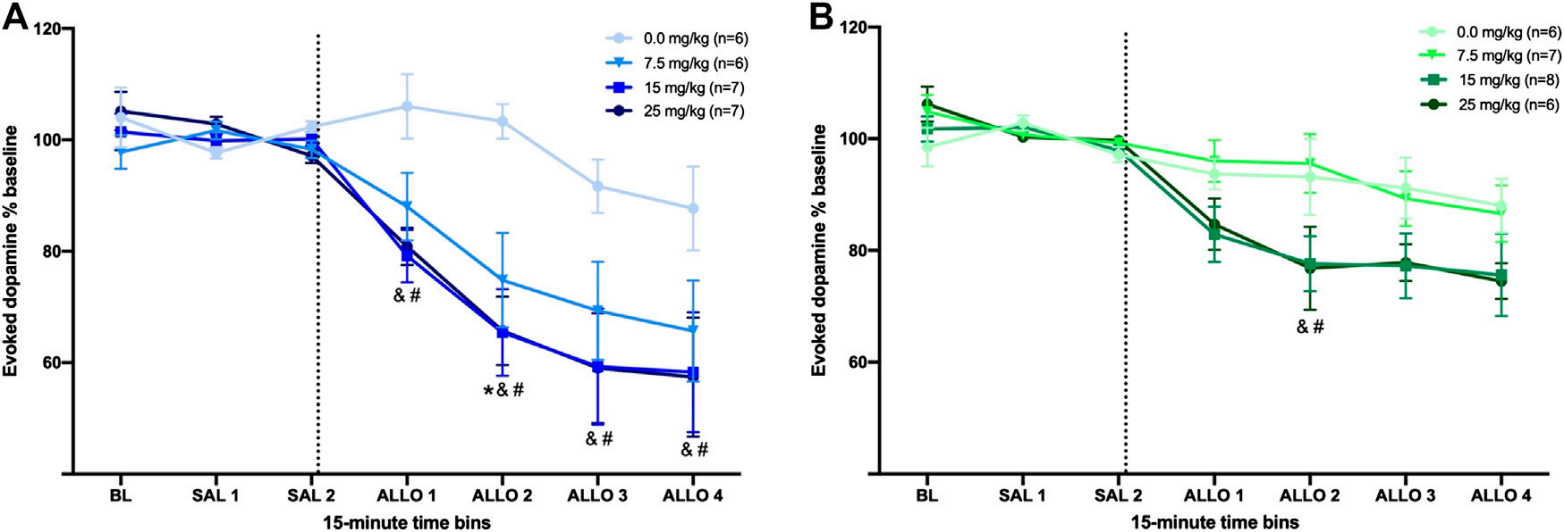
Allopregnanolone decreases evoked dopamine release in the nucleus accumbens of male and female rats. The dashed line indicates the moment when allopregnanolone (or vehicle) was injected. Data are normalized to the average [DA]_max_ after saline injections (SAL 1 and SAL 2); see methods for descriptions of time bins. **(A)** All three doses of allopregnanolone effectively decreased the evoked dopamine release into the NAc of male rats compared to vehicle group (between-subjects) and compared to the SAL 2 bin (within-subjects). *p < 0.05 7.5 mg/kg vs. vehicle; &*p* < 0.05 15 mg/kg vs. vehicle; #*p* < 0.05 25 mg/kg vs. vehicle (Vehicle, n = 6; 7.5 mg/kg, n = 6; 15 mg/kg, n = 7; 25 mg/kg, n = 7). **(B)** The higher doses of allopregnanolone reduced evoked dopamine transmission in females; all ALLO bins for the 15 and 25 mg/kg doses were significantly lower than SAL 2 bin (within-subjects), and ALLO 2 was significantly lower in 15 and 25 mg/kg groups compared to the vehicle group (between-subjects). &*p* < 0.05 15 mg/kg vs. vehicle; #*p* < 0.05 25 mg/kg vs. vehicle (Vehicle, n = 6; 7.5 mg/kg, n = 7; 15 mg/kg, n = 8; 25 mg/kg, n = 6).

**FIGURE 3:**
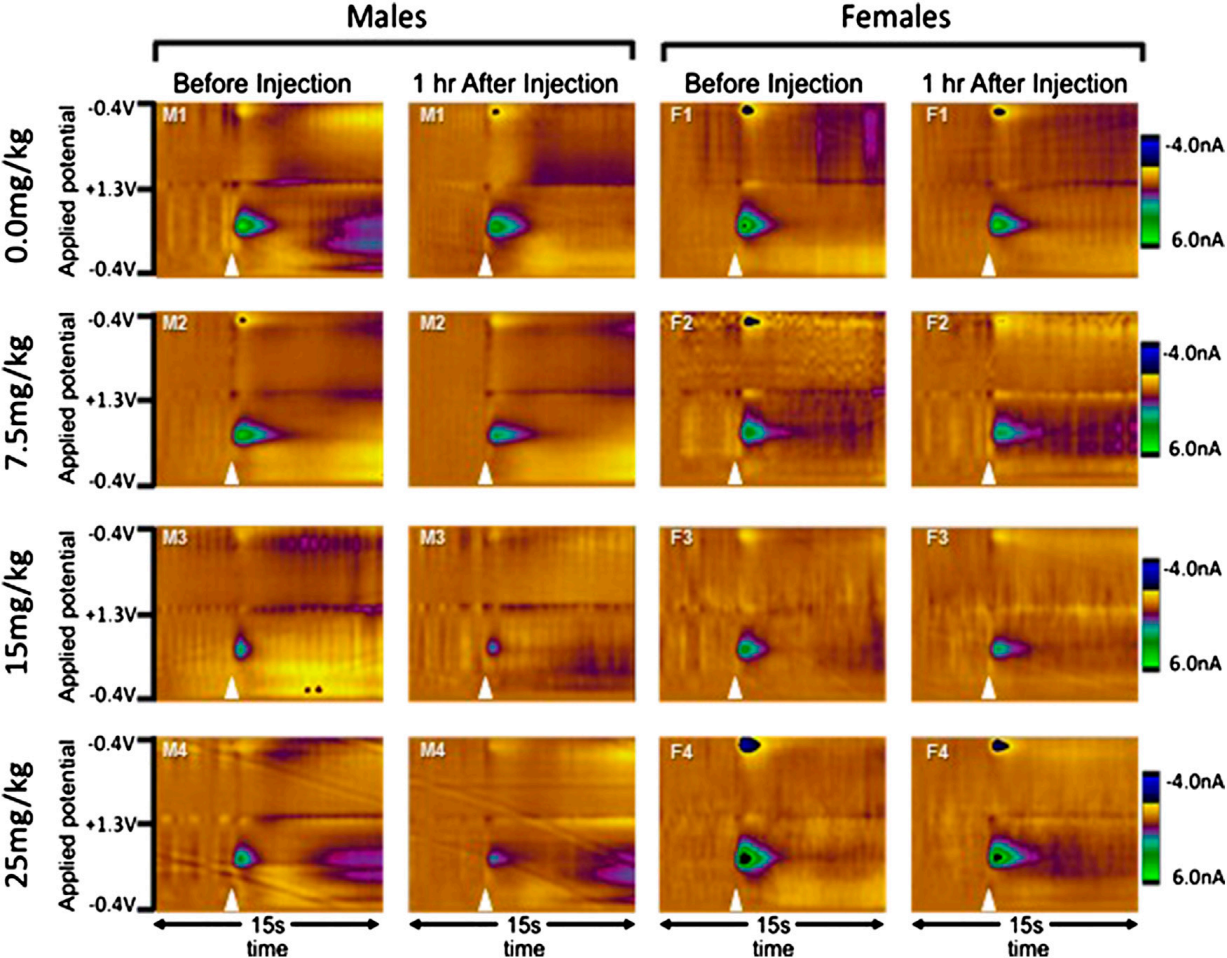
Examples of evoked dopamine release from individual rats before and after allopregnanolone. Electrochemical scans are shown from a single rat in each group (sex by dose) both before and one hour after the injection of 0.0 (vehicle), 7.5, 15, or 25 mg/kg allopregnanolone. Males are shown on the left and females on the right, with doses in ascending order from top to bottom. In the color plots, the oxidation and reduction currents are expressed in color and plotted by applied potential (y-axis) over time (x-axis). Electrical stimulation of the VTA is indicated by the white triangles, and oxidation of dopamine can be observed as changes in current at approximately 650 mV.

In male rats, all doses of allopregnanolone reduced [DA]_max_ as compared to the vehicle group, although the timing of this effect differed (Figure 2A). The two-way RM ANOVA yielded a significant interaction between dose and time (F_18,132_ = 2.7, $\eta_{p}^{2}=0.27$, *p* < 0.001), as well as main effects of dose (F_3,132_ = 4.0, $\eta_{p}^{2}=0.32$, *p* < 0.05) and time (F_6,132_ = 37.1, $\eta_{p}^{2}=0.63$, *p* < 0.001). Post-hoc analysis revealed that both 15 and 25 mg/kg allopregnanolone decreased [DA]_max_ within 15 min after injection when compared to the vehicle group (Allo 1: both *p* < 0.05), and this effect was maintained throughout the collection time (Allo 2: both *p* < 0.0005; Allo 3: both *p* < 0.005; Allo 4: both *p* < 0.01). In addition, 7.5 mg/kg allopregnanolone significantly reduced [DA]_max_ compared to the vehicle group during the Allo 2 bin (*p* < 0.05). We also made within-group comparisons of each bin to the Sal 2, the bin immediately before the allopregnanolone or vehicle injection. None of the timepoints differed in the vehicle group, while all post-injection timepoints differed from baseline in the 15 and 25 mg/kg groups (all *p*’s < 0.05), and the final three bins differed from baseline in the 7.5 mg/kg group (all *p*’s < 0.05). We also compared T½ across groups, and found that allopregnanolone did not change dopamine clearance; these data are reported in detail in the Supplementary Material. Representative dopamine signals obtained in male rats before and after each dose of allopregnanolone injection are shown in Figure 3.

As we observed that 7.5 mg/kg allopregnanolone significantly reduced evoked [DA]max, we conducted a follow-up experiment with a lower dose–5 mg/kg – in a separate group of male rats (n = 6). As described in the Supplementary Material, within-subject analysis confirmed that 5 mg/kg allopregnanolone reduced evoked dopamine release in the final three Allo bins compared to Sal 2 (one-way RM ANOVA: F_6,30_ = 11.1, $\eta_{p}^{2}=0.69$, *p* < 0.001), similar to the effect of 7.5 mg/kg dose.

In female rats, only the 15 and 25 mg/kg doses effectively reduced evoked dopamine release (Figure 2B). The two-way RM ANOVA of [DA]_max_ revealed a significant interaction between dose and time (F_18,138_ = 1.8, $\eta_{p}^{2}=0.19$, *p* < 0.05), as well as a main effect of time (F_6,138_ = 25.6, $\eta_{p}^{2}=0.53$, *p* < 0.001) and a marginal effect of dose (F_3,138_ = 2.5, $\eta_{p}^{2}=0.18$, *p* = 0.08). Post-hoc comparison confirmed that 15 and 25 mg/kg allopregnanolone decreased [DA]_max_ in the Allo 2 bin when compared to the vehicle group (both *p* < 0.05), while the 7.5 mg group did not differ from vehicle at any time. Likewise, [DA]_max_ in the vehicle and 7.5 mg allopregnanolone groups did not differ from the Sal 2 (the bin immediately preceding injection) at any time, while all post-injections timepoints were significantly reduced from baseline in the 15 and 25 mg/kg groups (all *p*’s < 0.05). Thus, while the 7.5 mg/kg dose effectively reduced dopamine release in males both within and between groups, it was ineffective in females. We also compared T½ across groups, and found that allopregnanolone did not change dopamine clearance; these data are reported in detail in the Supplementary Material. Representative dopamine signals obtained in female rats before and after each dose of allopregnanolone injection are shown in Figure 3.

### Females in Proestrus Are Less Responsive to Allopregnanolone Than Females in Other Days of the Estrous Cycle

**FIGURE 4:**
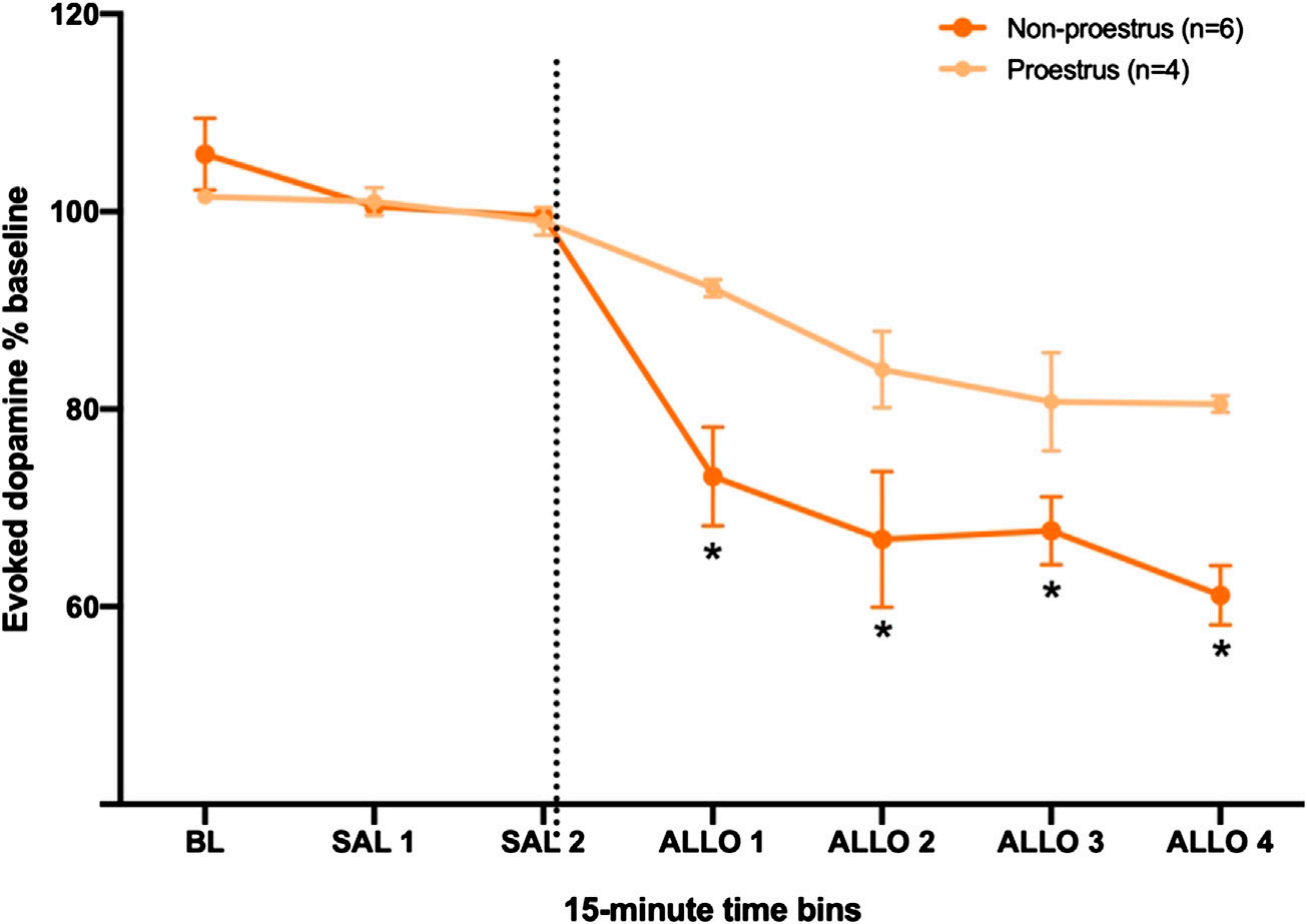
Females in proestrus (high progesterone) are less sensitive to allopregnanolone when compared to females in other estrous stages (low progesterone). The dashed line indicates the moment when allopregnanolone (15 or 25 mg/kg) was injected. Data are normalized to the average [DA]_max_ after saline injections (SAL 1 and SAL 2); see methods for descriptions of time bins. *p < 0.05 low vs. high progesterone levels (Low progesterone, n = 6; High progesterone, n = 4).

It is possible that the effect of allopregnanolone to decrease dopamine release in the NAc was affected by hormonal fluctuations during the estrous cycle. In rats, the estrous cycle includes proestrus, estrus, metestrus, and diestrus stages, and proestrus is the phase when the plasma levels of progesterone (a precursor to allopregnanolone) are highest (Smith et al., 1975). Moreover, studies have reported higher dopamine release during estrus (Xiao and Becker, 1994; Cummings et al., 2014; Calipari et al., 2017). Thus, we first compared baseline dopamine signals among females in proestrus (n = 7), estrous (n = 5) and metestus/diestrus (n = 7) in the subset of rats for which we determined cycle stage. Dopamine release was highest in rats in estrus, as compared to those in proestrus or metestus/diestrus, although this difference did not reach significance (H_2_ = 4.29, *p* < 0.12). We also found no significant difference in T½ across cycle stage; these data are reported in detail in the Supplementary Material. Next, we compared the effect of allopregnanolone on evoked dopamine in a subset of female rats that were assessed for cycle stage (Figure 4). Specifically, we pooled females from the 15 and 25 mg/kg dose groups, and compared rats that were confirmed to be in proestrus (high progesterone, n = 4) to those confirmed to be in diestrus, metestrus or estrus stages (low progesterone, n = 6). For this follow-up comparison, we did not include rats from the vehicle control group, as it contained rats that were in various stages of the estrous cycle, and we did not have sufficient numbers to separate them into proestrus and other stages. A two-way RM ANOVA yielded a significant interaction between time and cycle stage (F_6,42_ = 3.99, $\eta_{p}^{2}=0.36$, *p* < 0.01), as well as significant main effects of time (F_6,42_ = 33.40, $\eta_{p}^{2}=0.83$, *p* < 0.001) and cycle stage (F_1,42_ = 6.50, $\eta_{p}^{2}=0.37$, *p* < 0.05). Post-hoc comparisons showed that rats in proestrus were less sensitive to the allopregnanolone effect on dopamine transmission than rats in non-proestrus cycle stages at all post-injection times (Allo 1: *p* < 0.01; Allo 2: *p* < 0.01; Allo 3: *p* < 0.05; Allo 4: *p* < 0.01).

### Progesterone Decreased Evoked Dopamine Release Into the NAc of Male Rats

**FIGURE 5:**
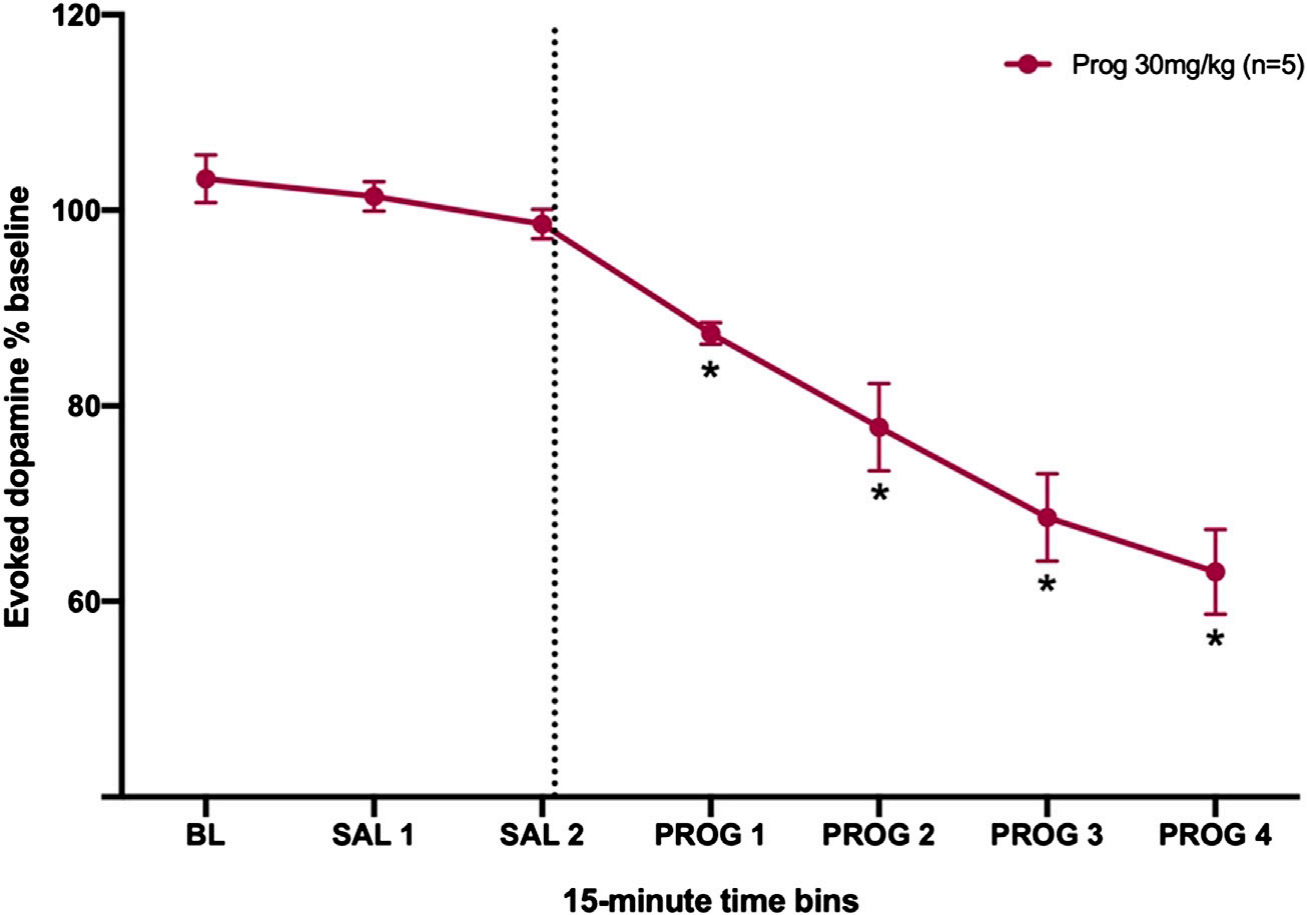
Progesterone decreases evoked dopamine release in the nucleus accumbens of male rats. The dashed line indicates the moment when the 30 mg/kg of progesterone was injected. *p < 0.05 vs. SAL 2 (n = 5).

As a proof of principle, to assess the effect of allopregnanolone’s precursor on dopamine transmission, we administered 30 mg/kg progesterone to male rats and measured evoked dopamine release in the NAc (Figure 5). Male rats were selected for this experiment due to their greater sensitivity to allopregnanolone as compared to females. For this proof-of-principle study, we did not run a separate vehicle control group, and instead used a within-subject analysis to assess progesterone effects. A one-way RM ANOVA confirmed that progesterone decreased evoked [DA]_max_ (F_6,24_ = 34.3, $\eta_{p}^{2}=0.90$, *p* < 0.001). When compared to Sal 2, [DA]_max_ in all post-injection time points was significantly reduced (post-hoc comparisons, all *p*’s < 0.05). Thus, administration of the precursor to allopregnanolone reduced evoked dopamine release similarly to allopregnanolone itself.

## Discussion

In the present study, we investigated the action of allopregnanolone on the mesolimbic dopamine system, and specifically on phasic fluctuations in dopamine release that are associated with motivated behavior (Schultz, 2016, Schultz, 2019). As allopregnanolone is inhibitory due to its allosteric actions at GABA_A_ receptors, and as other GABAergic compounds reduce evoked dopamine release (Gómez-A et al., 2017; Brodnik et al., 2019), we hypothesized that allopregnanolone would inhibit mesolimbic dopamine release. Our findings confirmed that allopregnanolone reduced electrically evoked dopamine release in the NAc of both male and female rats. Moreover, allopregnanolone action to reduce the evoked dopamine release was stronger in males than in females and stronger in females during the estrous stage characterized by low progesterone.

The ability of allopregnanolone to reduce evoked [DA]_max_ is likely to be due to its action at GABA_A_ receptors, although future studies are needed to confirm this hypothesis. This interpretation is supported by other studies showing that a variety of GABA agonists can reduce mesolimbic dopamine transmission (Smolders et al., 1995; Pitman et al., 2014; Gómez-A et al., 2017; Schelp et al., 2018; Brodnik et al., 2019). More specifically, allopregnanolone blunted dopamine release in an *ex vivo* slice preparation, an action prevented by the GABA_A_ receptor antagonist bicuculline (Knight et al., 2012). An open question is whether allopregnanolone regulates dopamine release via specific GABA_A_ receptor subtypes. While it is likely that the allopregnanolone effects on dopamine release reported here arise from neurosteroid action at GABA_A_ receptors, we did not confirm this with additional pharmacology. For example, one could block GABA_A_ transmission with bicuculline, which should also prevent any effect of allopregnanolone. However, manipulation of the GABA receptors *in vivo* would have broad effects and may act at different sites on the GABA receptor, both of which are likely to make its effect on the allopregnanolone response difficult to interpret. A separate but related limitation is that dopamine measurements in the present study were made in anesthetized rats, which may itself alter GABAergic dynamics and, by extension, allopregnanolone effects. Future studies can replicate this study in awake rats to confirm the sensitivity of male and female rats to allopregnanolone.

While GABA_A_ receptors are widely expressed throughout the brain, neurosteroids have greater affinity in some regions for receptors containing the δ subunit (for review, see Belelli and Lambert, 2005), which occur extrasynaptically and have been shown to mediate GABAergic tonic inhibition in different brain regions (Carver et al., 2014; Marowsky and Vogt, 2014), including the VTA (Vashchinkina et al., 2014). While systemic allopregnanolone can produce conditioned place preference (Finn et al., 1997), intracerebroventricular infusion of allopregnanolone produced conditioned place aversion in rats (Beauchamp et al., 2000), and it was shown that this effect was dependent on δ subunits in the VTA (Vashchinkina et al., 2014). Intracerebroventricular allopregnanolone also prevented the increase in extracellular dopamine concentrations in the NAc induced by foot-shock (Motzo et al., 1996). The NAc is also rich in GABA_A_ δ-containing receptors (Maguire et al., 2014). Thus, δ-containing GABA_A_ receptors in the VTA and NAc may regulate mesolimbic dopamine release and participate in the effects of allopregnanolone observed in our study, although future experiments are required to confirm a role of δ subunits. For instance, genetic models such as knock-out mice can be used to determine whether allopregnanolone affects dopamine release through a specific receptor subtype.

We observed that allopregnanolone reductions in evoked dopamine release were more robust in males than in females. Specifically, all doses of allopregnanolone (7.5, 15, and 25 mg/kg) reduced evoked dopamine release compared to vehicle in males, while only the two higher doses reduced evoked dopamine release compared to vehicle controls in females. Other studies confirm similar sex differences in allopregnanolone action; for example, females that received allopregnanolone were less sedated and recovered more rapidly from the neurosteroid effect than males at the same dose (Irwin et al., 2015). Similarly, the α4β3δ GABA_A_ receptor agonist THIP induced significantly greater tonic current in the substantia nigra pars reticulata neurons of young male rats than of females at the same age (Chudomel et al., 2015). We can speculate that the sex variance on the inhibitory action of allopregnanolone could be explained, at least in part, by a greater expression of GABA_A_ receptors in males compared to females (Juptner and Hiemke, 1990). Alternatively, circulating progesterone in cycling females may also contribute to the sex difference, as investigated below.

We found that estrous cycle in females influenced sensitivity to allopregnanolone action. Relevant to this study, there are two peaks of progesterone secretion from the ovaries into blood circulation during the estrous cycle. The first peak of progesterone is small and occurs from the newly formed corporea lutea during the afternoon of metestrus, while the second and larger peak of progesterone arises from the granulosa cells of the preovulatory follicle at proestrous (Nequin et al., 1979; Freeman, 2006). Here we observed that during proestrus, when the circulating levels of progesterone are highest, evoked dopamine release was less affected by allopregnanolone than during other phases of the cycle. Progesterone may directly influence dopamine release by inducing dephosphorylation and inactivation of tyrosine hydroxylase in dopamine neurons (Arbogast and Voogt, 2002), a limiting step in dopamine synthesis, which may result in less dopamine available for release. However, in the present study we did not observe significant differences in baseline dopamine release between females in proestrus versus other stages of the estrous cycle with lower progesterone levels (see Supplementary Material), suggesting that any changes in synthesis did not impact readily-releasable dopamine. Indeed, reported changes in dopamine release across the estrous cycle are tied to fluctuations in estrogen rather than progesterone (Xiao and Becker, 1994; Cummings et al., 2014; Calipari et al., 2017). One straightforward explanation of the present data is that higher levels of circulating progesterone would lead to higher levels of GABAergic metabolites allopregnanolone and THDOC, rendering exogenous allopregnanolone less effective to further alter dopamine release. Moreover, in addition to oscillations in the availability and synthesis of neurosteroids across the ovarian cycle, the expression of δ-containing GABA_A_ receptors in multiple brain regions also fluctuates across the ovarian cycle (Griffiths and Lovick, 2005; Lovick et al., 2005; Maguire et al., 2005; Hantsoo and Epperson, 2020). Such fluctuation may contribute to observations in women across the menstrual cycle; for example, high levels of circulating progesterone during the luteal phase of the menstrual cycle was shown to decrease stress-induced cocaine craving and anxiety (Sinha et al., 2007), phenotypes linked to dopamine and GABA, respectively.

Similarly to allopregnanolone, progesterone reduced evoked dopamine release in males, which is consistent with conversion to its metabolite allopregnanolone. A caveat is that this was a “proof of principle” study and was missing a separate vehicle group. Other researchers have demonstrated that progesterone modulates several types of receptors, but can just activate the GABA_A_ receptor after its conversion to allopregnanolone or other GABAergic metabolites (Callachan et al., 1987). Similar to allopregnanolone, progesterone can be dysregulated in psychiatric disorders (for review, see Bristot et al., 2014). For example, post-traumatic stress disorder in women is associated with a block in conversion of progesterone to allopregnanolone (Pineles et al., 2018). Moreover, progesterone administration to cocaine-dependent subjects increased allopregnanolone in plasma (Milivojevic et al., 2019), normalized cortisol levels, improved mood, and reduced cocaine craving (Milivojevic et al., 2016). Thus, progesterone can elevate allopregnanolone levels in humans, highlighting both neurosteroids as potentially important clinical therapeutics.

The data reported here suggest that females are less sensitive to allopregnanolone regulation of dopamine release, particularly when females are in proestrus, the phase of the rodent cycle accompanied by the highest surge in progesterone. We speculate that higher levels of circulating progesterone, and therefore higher levels of endogenous progesterone metabolites allopregnanolone and THDOC, underlies the lower sensitivity to the effects of exogenous allopregnanolone, due to observations that these neurosteroids can down-regulate various GABA_A_ receptors (Concas et al., 1998; Smith et al., 1998; Belelli and Lambert, 2005, Smith et al., 2007; Wang, 2011). Indeed, THDOC can also regulate dopamine release (Grobin et al., 1992). However, these effects will need to be confirmed in multiple ways. First, the estrous cycle data were determined by a one-time inspection of vaginal cell morphology. A better determination of cycle stage would include assessment over multiple days to track the cycle, blood estradiol and progesterone levels, or ovarian morphology. This would allow full dose-response curves of allopregnanolone and other neuroactive steroids to be conducted in rats at different stages of the estrous cycle. Second, the hypothesis that higher circulating levels of progesterone elevate allopregnanolone levels in blood and/or brain will need to be tested in both in intact, cycling females, and in ovariectomized females administered physiological levels of progesterone. A third area of inquiry would be to study the interaction of progesterone, allopregnanolone and estradiol across the rodent estrous cycle, as estradiol is also known to enhance dopamine release (Xiao and Becker, 1994).

It is known that deficits in GABAergic neurotransmission may contribute to some psychiatric disorders that often include dysregulation of dopamine transmission, such as depression, anxiety, and addiction (Mohler, 2012; Stephens et al., 2017; Duman et al., 2019). Neurosteroids such as allopregnanolone are a promising approach to modulate GABAergic systems to treat psychiatric disorders and the present study extends our understanding of the neuropharmacology of exogenous neurosteroid administration.

## Data Availability Statement

The raw data supporting the conclusions of this article will be made available by the authors, without undue reservation.

## Ethics Statement

The animal study was reviewed and approved by Institutional Animal Care and Use Committee of University of North Carolina at Chapel Hill.

## Author Contributions

AD: Project administration; Investigation; Validation; Formal analysis; Writing-Original Draft; Writing-Review and Editing GM: Project administration; Investigation; Formal analysis; Writing-Review and Editing MM: Investigation; Formal analysis; Writing-Original Draft; Writing-Review and Editing AG-A: Supervision; Investigation; Writing-Review and Editing TO’B: Resources; Writing-Review and Editing CC: Funding acquisition; Conceptualization; Writing-Review and Editing AM: Funding acquisition; Conceptualization; Resources; Writing - Review and Editing DR: Funding acquisition; Conceptualization; Formal analysis; Writing-Original Draft Writing-Review and Editing.

## Funding

This research was funded by NIH NC TraCS UL1TR001111 project 2KR1011813, NIH P60 AA011605, a Foundation of Hope (Raleigh, NC, United States) research award, and Bowles Center for Alcohol Studies at the University of North Carolina. MHM was supported on “UNC PREP in the Biomedical Sciences” (NIH R25 GM089569). AD and GM were supported on Postdoctoral Program Abroad Fellowships (CAPES)—Coordination of Improvement of Higher Education Personnel (Brazil).

## Conflict of Interest

The authors declare that the research was conducted in the absence of any commercial or financial relationships that could be construed as a potential conflict of interest.

## **Abbreviations:**

AP, anterior-posterior; [DA]_max_, maximum dopamine concentration; DV, dorsal-ventral; GABA, γ-aminobutyric acid; ML, medial-lateral; NAc, nucleus accumbens; VTA, ventral tegmental area
