# Supplementary material for: Allopregnanolone Decreases Evoked Dopamine Release Differently in Rats by Sex and Estrous Stage
Source: Front Pharmacol. 2021 Jan 14;11:608887. doi: 10.3389/fphar.2020.608887 (PMC7840599; doi:10.3389/fphar.2020.608887)
Supplement: Supplementary file 1 [file datasheet1.docx]

Supplemental Material for Dornellas, Macedo et al., “Allopregnanolone decreases evoked dopamine release differently in rats by sex and estrous stage,” *Neuropharmacology*

***Baseline evoked dopamine by sex***

To analyze if baseline dopamine signals diverged between males and females, we pooled the 9 evoked dopamine signals prior to the injection of allopregnanolone or vehicle. We performed this analysis on the dopamine concentration (not normalized), as this analysis focused on absolute differences in concentration rather than relative changes in signal due to neurosteroids. Evoked [DA]_max_ was slightly higher in females (0.76 ± 0.08 nM, range 0.34 – 1.76) than in males (0.60 ± 0.066 nM, range 0.23 – 1.45), a difference that was marginally significant (Mann-Whitney U = 254, p > 0.08). We next compared dopamine clearance, as indicated by T½ (the time for dopamine to clear to half maximum concentration). T½ was similar between male (0.86 ± 0.22 seconds) and female (0.87 ± 0.24 seconds) rats at baseline (t_49_=0.233, p>0.81).

Sex differences in dopamine release have been previously reported, such as reduced extracellular dopamine concentrations measured with microdialysis in ovariectomized females compared to castrated males (Castner et al., 1993; Xiao and Becker, 1998; Cummings et al., 2014). Moreover, fast scan cyclic voltammetry was used to determine that females exhibit greater phasic dopamine release and faster uptake in the caudate nucleus than males (Walker et al., 2006; Walker et al., 2000), although this did not always translate to higher evoked [DA]_max_ (Walker et al., 2006), as [DA]_max_ is influenced by both release and uptake mechanisms. Our finding that [DA]_max_ was marginally higher in females than males is consistent with this literature.

***Baseline evoked dopamine by estrous cycle***

**
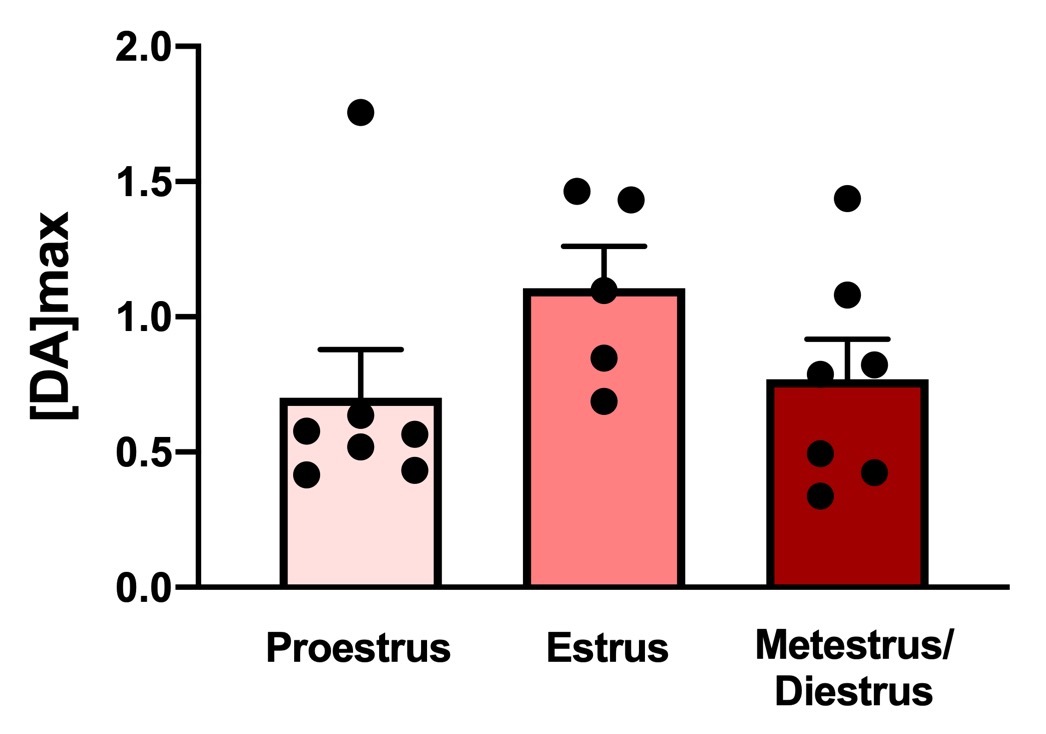
Supplemental Figure 1.** Evoked dopamine release at baseline did not significantly vary across the estrous cycle in rats assessed for cycle stage.

We also determined whether estrous cycle stage – specifically proestrus, which is associated with high circulating levels of progesterone (Smith et al., 1975) – affected evoked dopamine release in the subset of rats assessed for cycle stage (n=19). Specifically, we compared [DA]_max_ in the 9 evoked dopamine signals prior to the injection of allopregnanolone or vehicle among females in proestrus (n=7), estrus (n=5) and metestus/diestrus (n=7). As expected from the literature, [DA]_max_ was highest in estrus as compared to other cycle stages (**Supplemental Figure 1**) , although this difference did not reach statistical significance (Kruskal-Wallis one-way ANOVA on ranks, H_2_=4.29, p<0.12). We next compared dopamine clearance. At baseline, T½ was similar across female rats in proestrus, estrus and metestrus/diestrus (Kruskal-Wallis one-way ANOVA on ranks, H_2_=1.44, p<0.49).

We did not see baseline differences in [DA]_max_ among females in proestrus (high progesterone) compared to other stages. This outcome is not unsurprising, given that while dopamine dynamics are known to change across the estrous cycle, this is primarily due to estradiol effects (Xiao and Becker, 1998; Cummings et al., 2014; Calipari et al., 2017) rather than progesterone. Nevertheless, this effect is not always observed: Walker et al (2000) did not detect differences in evoked dopamine release or in V_max_ or K_m_ of the dopamine transporter across the estrous cycle. In the present study, the average [DA]_max_ was highest in rats assessed during estrus when levels of circulating estrogen are highest. While this effect did not reach significance, it may be due to the low number of rats assessed in estrus. Alternatively, it is possible that females categorized in one stage were actually in transition to or from that stage; future studies can directly measure circulating estrogen and progesterone levels to correlate circulating hormone levels with dopamine dynamics.

**Supplemental Figure 2.** Dopamine clearance (T½) is not affected by allopregnanolone administration. Across the recording, T½ slowed by approximately 15% in (A) male and (B) female rats, with no difference among doses of allopregnanolone. S = Sal 2 bin; A = Allo 4 bin.

**Supplemental Table 1.** Dopamine clearance (T½) increases over time in male and female rats and is not affected by allopregnanolone administration. Values are expressed as mean ± SEM.

|  | **Males** | | | **Females** | | | |
| --- | --- | --- | --- | --- | --- | --- | --- |
| **Dose** | *N* | *Sal 2* | *Allo 4* | | *N* | *Sal 2* | *Allo 4* |
| *0.0 mg/kg* | 6 | 0.87 ± 0.04 | 0.93 ± 0.09 | | 6 | 0.93 ± 0.05 | 1.05 ± 0.06 |
| *7.5 mg/kg* | 5 | 1.03 ± 0.13 | 1.23 ± 0.15 | | 7 | 1.01 ± 0.11 | 1.12 ± 0.17 |
| *15 mg/kg* | 6 | 0.73 ± 0.09 | 0.87 ± 0.14 | | 8 | 0.81 ± 0.10 | 0.94 ± 0.08 |
| *25 mg/kg* | 7 | 0.83 ± 0.09 | 0.97 ± 0.09 | | 6 | 0.73 ± 0.06 | 0.89 ± 0.06 |

***No effect of allopregnanolone on clearance of evoked dopamine in the NAc in male and female rats***

We analyzed whether allopregnanolone altered dopamine clearance, as changes in both dopamine release and clearance could contribute to changes in [DA]_max_. Clearance half-life (T½), the time required for dopamine concentration to decrease to half of [DA]_max_ (Yorgason et al., 2011), was taken as a measurement of dopamine clearance rate. We analyzed clearance in the Sal 2 bin (the 15 min immediately prior to allopregnanolone or vehicle injection) and in the Allo 4 bin (the final 15 min of data collection). Rats (n = 2 males) were excluded from analysis because they exhibited dopamine currents in some files that did not clear to <50% of the peak amplitude.

Allopregnanolone did not alter dopamine clearance (T½); however, T½ increased by approximately 15% over time (from Sal 2 to Allo 4 bins) across all groups of male and female rats (**Supplemental Table 1** and **Supplemental Figure 2**). A two-way RM ANOVA yielded a significant main effect of time for males (F_1,20_ = 9.668, p<0.05) and females (F_1,23_ = 9.571, p<0.05), but no significant effect of dose or interaction between the two. Slower clearance over time is a common observation (Shnitko and Robinson 2014; Ng et al., 1992; Shnitko et al., 2014), which is likely not due to urethane anesthesia (Brodnik and España, 2015), but rather to changes on the surface of the carbon-fiber electrode caused by biofouling that result in slower signal diffusion (Venton and Cao, 2020). These data support the interpretation that the reduction in [DA]_max_ after allopregnanolone administration is due to reduced dopamine release rather than faster dopamine clearance.

***5 mg/kg allopregnanolone reduced evoked dopamine release in male rats***

In male rats, we observed that 7.5 mg/kg allopregnanolone significantly reduced evoked [DA]_max_. Therefore, to extend the dose-response curve, we conducted a follow-up experiment with 5 mg/kg allopregnanolone in a separate group of male rats (n=6). As shown in **Supplemental Figure 3**, within-subject analysis confirmed that 5 mg/kg allopregnanolone reduced evoked dopamine release (one-way RM ANOVA: F_6,30_=11.1, p<0.001). Holm-Sidak post-hoc comparisons revealed that [DA]_max_ in the final three Allo bins were significantly less than in Sal 2 (Allo 2: p<0.05; Allo 3: p<0.05; Allo 4: p<0.05). Because these data were collected several months after the data in Figure 1 of the main manuscript, we do not statistically compare it to the original data, but we overlaid them for comparison purposes in **Supplemental Figure 3**. These findings reinforce the conclusion that males are sensitive to the effect of allopregnanolone to reduce evoked dopamine release, and inform future dose-response experiments in adult male rats.

**Supplemental Figure 3.** Follow-up experiment: 5 mg/kg allopregnanolone reduced evoked dopamine release in male rats. (Top) 5 mg/kg allopregnanolone significantly decreased [DA]max 15 – 60 min after administration (* p < 0.05 versus SAL 2). (Bottom) The same data were graphed with the larger dose response curve (Fig. 1A of the main manuscript) to establish the relative dose-dependent reductions. However, as the 5 mg/kg data were collected several months after the other doses, we do not statistically compare it to the original data.

***Lack of 7.5 mg/kg allopregnanolone effect on evoked dopamine release in female rats was not dependent on estrous cycle stage***

In female rats, 7.5 mg/kg allopregnanolone did not reduce evoked dopamine release, as [DA]_max_ in that group was no different than vehicle. However, we found that rats in proestrus were less sensitive to 15 and 25 mg/kg allopregnanolone. Thus, it is possible that this lack of effect of 7.5 mg/kg was due to many rats in proestrus. In this group, 2 rats were in proestrus, 4 were in other cycle stages, and one was not assessed. We compared the 2 proestrus rats with the 4 non-proestrus rats with a 2-way RM ANOVA, and the two groups did not differ (no main effect of group: F_1,24_=2.4, p>0.19; group by time interaction: F_6,24_=1.2, p>0.32). There was a main effect of time (F_6,24_=2.9, p<0.05), reflecting a downward trend for lower [DA]_max_ over the recording, but post-hoc comparisons revealed that no time bins were significantly different from the SAL 2 bin immediately preceding allopregnanolone administration. While this comparison is underpowered, with only 2 rats in the proestrus stage, it suggests that the lack of effect of 7.5 mg/kg was not due to oversampling rats in proestrus.

**Supplemental References**

Brodnik, Z.D., & España, R.A., 2015. Dopamine uptake dynamics are preserved under isoflurane anesthesia. *Neurosci Let* 606, 129–134.

Calipari, E.S., Juarez, B., Morel, C., Walker, D.M., Cahill, M.E., Ribeiro, E., Roman-Ortiz, C., Ramakrishnan, C., Deisseroth, K., Han, M.H., Nestler, E.J., 2017. Dopaminergic dynamics underlying sex-specific cocaine reward. *Nat Commun* 8, 13877.

Castner, S.A., Xiao, L., Becker, J.B., 1993. Sex differences in striatal dopamine: in vivo microdialysis and behavioral studies. *Brain research* 610, 127-134.

Cummings, J.A., Jagannathan, L., Jackson, L.R., Becker, J.B., 2014. Sex differences in the effects of estradiol in the nucleus accumbens and striatum on the response to cocaine: neurochemistry and behavior*. Drug Alcohol Depend* 135, 22-28.

Ng, J.P., Menacherry, S.D., Liem, B.J., Anderson, D., Singer, M., & Justice, J.B., 1992. Anomalous effect of mazindol on dopamine uptake as measured by in vivo voltammetry and microdialysis. *Neurosci Let* 134(2), 229–232.

Shnitko, T.A., & Robinson, D.L., 2014. Anatomical and pharmacological characterization of catecholamine transients in the medial prefrontal cortex evoked by ventral tegmental area stimulation. *Synapse* 68(4), 131–143.

Shnitko, T.A., Kennerly, L.C., Spear, L.P., & Robinson, D.L., 2014. Ethanol reduces evoked dopamine release and slows clearance in the rat medial prefrontal cortex. *Alcohol Clin Exp Res* 38(12), 2969–2977.

Smith, M.S., Freeman, M.E., Neill, J.D., 1975. The control of progesterone secretion during the estrous cycle and early pseudopregnancy in the rat: prolactin, gonadotropin and steroid levels associated with rescue of the corpus luteum of pseudopregnancy. *Endocrinology* 96, 219-226.

Venton, B.J., & Cao, Q., 2020. Fundamentals of fast-scan cyclic voltammetry for dopamine detection. *Analyst* 145(4), 1158–1168.

Walker, Q.D., Ray, R., Kuhn, C.M., 2006. Sex differences in neurochemical effects of dopaminergic drugs in rat striatum. *Neuropsychopharmacology* 31, 1193-1202.

Walker, Q.D., Rooney, M.B., Wightman, R.M., Kuhn, C.M., 2000. Dopamine release and uptake are greater in female than male rat striatum as measured by fast cyclic voltammetry. *Neuroscience* 95, 1061-1070.

Xiao, L., Becker, J.B., 1998. Effects of estrogen agonists on amphetamine-stimulated striatal dopamine release. *Synapse* 29, 379-391.

Yorgason, J.T., España, R.A., & Jones, S.R., 2011. Demon voltammetry and analysis software: analysis of cocaine-induced alterations in dopamine signaling using multiple kinetic measures. *J Neurosci Methods* 202(2), 158–164.
